# Machine learning analysis reveals abnormal functional network hubs in the primary angle-closure glaucoma patients

**DOI:** 10.3389/fnhum.2022.935213

**Published:** 2022-08-24

**Authors:** Ri-Bo Chen, Yu-Lin Zhong, Hui Liu, Xin Huang

**Affiliations:** ^1^Department of Radiology, Jiangxi Provincial People’s Hospital, The First Affiliated Hospital of Nanchang Medical College, Nanchang, China; ^2^Department of Ophthalmology, Jiangxi Provincial People’s Hospital, The First Affiliated Hospital of Nanchang Medical College, Nanchang, China

**Keywords:** PACG, degree centrality, fMRI, SVM, brain network

## Abstract

**Background:**

Primary angle-closure glaucoma (PACG) is a serious and irreversible blinding eye disease. Growing studies demonstrated that PACG patients were accompanied by vision and vision-related brain region changes. However, whether the whole-brain functional network hub changes occur in PACG patients remains unknown.

**Purpose:**

The purpose of the study was to investigate the brain function network hub changes in PACG patients using the voxel-wise degree centrality (DC) method.

**Materials and methods:**

Thirty-one PACG patients (21 male and 10 female) and 31 healthy controls (HCs) (21 male and 10 female) closely matched in age, sex, and education were enrolled in the study. The DC method was applied to investigate the brain function network hub changes in PACG patients. Moreover, the support vector machine (SVM) method was applied to distinguish PACG patients from HC patients.

**Results:**

Compared with HC, PACG patients had significantly higher DC values in the right fusiform, left middle temporal gyrus, and left cerebelum_4_5. Meanwhile, PACG patients had significantly lower DC values in the right calcarine, right postcentral gyrus, left precuneus gyrus, and left postcentral gyrus. Furthermore, the SVM classification reaches a total accuracy of 72.58%, and the ROC curve of the SVM classifier has an AUC value of 0.85 (*r* = 0.25).

**Conclusion:**

Our results showed that PACG patients showed widespread brain functional network hub dysfunction relative to the visual network, auditory network, default mode network, and cerebellum network, which might shed new light on the neural mechanism of optic atrophy in PACG patients.

## Introduction

Primary angle-closure glaucoma (PACG) is a serious and irreversible blinding eye disease worldwide. Angle closure is a key pathological process in PACG patients. The main clinical manifestations of PACG are elevated intraocular pressure, eye pain, and vision loss. There are multiple risk factors for PACG, such as genetic predisposition (Wiggs and Pasquale, 2017), hypertension (Lowry and Sanders, 2021), diabetes mellitus (Song et al., 2016), and autonomic dysfunction (Na et al., 2010). Elevated intraocular pressure leads to retinal ganglion cell apoptosis in PACG patients, which is an important pathological feature of PACG. Visual field examination is important for the assessment of visual function in glaucoma patients (De Moraes et al., 2017). However, recent studies have shown that glaucoma causes optic nerve atrophy as well as trans-synaptic degeneration of visual pathways (Lawlor et al., 2018).

Magnetic resonance imaging (MRI) techniques have been successfully applied to identify changes in neural function and structure within the visual cortex of glaucoma patients. Zhou et al. (2017) demonstrated an expanded representation of parafoveal areas in the visual cortex of primary open-angle glaucoma (POAG) patients, compared with healthy controls (HCs). Duncan et al. (2012) demonstrated that POAG patients had differences in cerebral blood flow (CBF) between ventral V1 and dorsal V1. These differences were correlated with visual function in the superior and inferior visual fields. Wang et al. (2017) reported that the POAG patients had decreased functional connectivity within the visual network. Moreover, glaucoma patients exhibited structural changes in the brain. Using diffusion kurtosis imaging, Li et al. (2020) demonstrated that patients with normal-tension glaucoma had microstructural abnormalities in bilateral BA17, BA18, and BA19. Hernowo et al. (2011) found reduced visual pathway volumes in glaucoma patients; affected areas included the optic nerves, optic chiasm, optic tract, and optic radiations. The above studies indicated that patients with glaucoma have functional and structural changes in the visual pathways and visual cortex. To our knowledge, there have been few studies of whole-brain network changes in PACG patients.

The human brain is a complex functional connectome (Sporns, 2011) that plays important roles in physiological processes such as visual function (Wang et al., 2018) and auditory function (Gurtubay-Antolin et al., 2021). Previous neuroimaging studies demonstrated that brain functional network hubs are involved in the integration of information from network elements. Hubs enable the integration of diverse sources of information; they also balance opposing pressures during the evolution of segregated networks. Increased degree centrality (DC) values indicate the enhanced local network information transmission. Meanwhile, the decreased DC values indicate the impaired local network information transmission. The voxel-wise DC method is a graph-based technique that measures the functional relationships of specific voxels throughout the connectivity matrix of the brain, rather than in specific nodes or networks (Zuo et al., 2012). The advantage of the DC method is that the functional network of the whole brain voxel can be calculated without presupposition experimental hypothesis. Machine learning methods combined with the fMRI method have provided a systematic approach for developing an automatic, objective, and sophisticated classification of diseases. The support vector machine (SVM) method is the most commonly used supervised machine learning algorithm for MRI classification that enables individual-level classification and detects biomarkers on the basis of neuroimaging data. Glaucoma is not only associated with functional abnormalities in the visual area but has also been associated with neurodegenerative diseases. However, the effects of optic atrophy on whole-brain network hubs in PACG patients remain unclear. Thus, we hypothesized that PACG patients are accompanied by brain functional network hub changes.

Based on this hypothesis, the present study was performed to determine whether PACG patients exhibited abnormal changes in brain function network hubs. Moreover, the SVM method was used to assess classification power using the DC map as a feature.

## Materials and methods

### Participants

In total, thirty-one patients with PACG (21 males and 10 females) were enrolled from the Department of Ophthalmology, Jiangxi Provincial People’s Hospital. The diagnostic criteria of PACG were: (1) the intraocular pressure was greater than 21 mmHg in both eyes; (2) optic disc/cup area > 0.6; (3) typical vision field defect (paracentric obscura, arcuate obscura, nasal ladder, fan-shaped field defect, and peripheral field defect); (4) without any other ocular diseases.

The exclusion criteria of PACG were: (1) advanced PACG patients are associated with severe eye pain; (2) PACG patients with a history of surgery; (3) PACG patients with glaucoma-related eye complications, including neovascular glaucoma, high myopia, optic neuritis, cataracts, eye atrophy, and corneal edema; and (4) PACG patients with psychiatric disorders, brain trauma, and other diseases.

Thirty-one HCs (21 males and 10 females) were also recruited for this study. The inclusion criteria were: (1) without any ocular disease with uncorrected visual acuity (VA) > 1.0; 2) no cardiovascular system diseases; and no psychiatric disorders.

#### Ethical statement

All research methods followed the Declaration of Helsinki and were approved by the Ethical Committee for Medicine of Jiangxi Provincial People’s Hospital. Participants enrolled in the study of their own accord and were informed of the purpose, methods, as well as potential risks before signing an informed consent form.

### Clinical evaluation

The VA of all subjects was measured by using the logMAR table and Intraocular pressure was assessed by using automatic intraocular pressure measurement. The best-corrected VA of both eyes was measured in each group.

### Magnetic resonance imaging data acquisition

MRI scanning was performed on a 3-Tesla MR scanner (750W GE Healthcare, Milwaukee, WI, United States) with an eight-channel head coil. All participants were required to close their eyes without falling asleep when undergoing MRI scanning. The subjects should be kept calm and not engaged in specific thoughts.

### Data pre-processing

All preprocessing was performed using the toolbox for Data Processing & Analysis of Brain Imaging (DPABI)^1^ (Yan et al., 2016) and briefly following the steps: (1) the first ten volumes of BOLD maps were removed. (2) Slice timing and head motion correction were conducted. (4) Normalized fMRI data were re-sliced with a resolution of 3 × 3 × 3 mm^3^. (5) Data detrends; (6) Linear regression analysis was applied to regress out several covariates (mean framewise displacement, global brain signal, and averaged signal from cerebrospinal fluid and white matter). (7) Temporal band-pass filtering was performed (0.01–0.08 Hz).

### Voxel-wise degree centrality analysis

According to previous studies, we analyzed the different degree of centrality values with a correlation threshold of (*r* = 0.15, 0.2, 0.25, 0.3) in this study (Zuo et al., 2012). The voxel-wise functional network was generated for each subject, for which we took each voxel as a node and inter-voxel correlations as the edge. Within the default brain mask provided by DPABI (in the MNI-152 standard space with 3 × 3 × 3 mm^3^ voxel size and resolution of 61 × 73 × 61), we used the preprocessed functional images to perform a voxel-wise correlation analysis. From the *n* × *n* Pearson’s correlation coefficient matrix, a map of the degree of the connectivity was computed by counting for each voxel the number of voxels it was correlated to above a threshold of *r* > 0.25. A high threshold was chosen to eliminate counting voxels that had low temporal correlation attributable to signal noise. The *z*-score map was smoothed with a 6-mm full-width-half-maximum Gaussian kernel.

### Support vector machine analysis

The SVM algorithm was performed by using the Pattern Recognition for Neuroimaging Toolbox (PRoNTo) software Cyclotron Research Centre, University of Liège, Belgium (Schrouff et al., 2013). The following steps were followed: (1) the DC maps (*r* = 0.15, 0.2, 0.25, 0.3) were used as classification feature. (2) Then, the leave-one-out cross-validation (LOOCV) technique was applied to classifier validation. (3) The total accuracy, specificity, sensitivity, and area under the receiver operating characteristic curve (AUC) were calculated.

### Statistical analysis

The chi-square test was used to calculate the sex and handness and independent sample *t*-test was used for age, education, and BCVA between the two groups.

The one-sample *t*-test was conducted to assess the group mean of DC maps (*r* = 0.15, 0.2, 0.25, 0.3). The two-sample *t*-test was used to compare the two group differences in the DC maps (*r* = 0.15, 0.2, 0.25, 0.3) using the Gaussian random field (GRF) method. (two-tailed, voxel-level *P* < 0.01, GRF correction, cluster-level *P* < 0.05).

## Results

### Demographics and disease characteristics

There were no statistically significant differences between the PACG and HC groups in gender, education, or age, but significant differences in BCVA of right eye (*p* < 0.001), left eye (*p* < 0.001). The results of these data are listed in Table 1.

**TABLE 1:** Demographic and clinical measurements between patients with PACG and HCs.

Condition	PACG group	HC group	*T*-value	*P*-value
Age (years)	50.96 ± 4.85	50.82 ± 6.76	0.727	0.470
Sex (male/female)	21/10	21/10	<0.001	1.000
Handness (right/left)	29/2	28/3	0.218	0.641
Education (years)	12.61 ± 5.88	11.46 ± 6.86	0.670	0.506
BCVA-OD	0.15 ± 0.10	1.18 ± 0.12	0.626	<0.001
BCVA-OS	0.30 ± -0.12	1.14 ± 0.10	0.538	<0.001

Chi-square test for sex and handness. An independent t-test was used for age, education, and BCVA. Data are displayed as mean ± SD.

HC, healthy control; BCVA, best-corrected visual acuity; OD, oculus dexter; OS, oculus sinister; N/A, not applicable; R, right.

### Different degree centrality between primary angle-closure glaucoma and healthy control group

**FIGURE 1:**
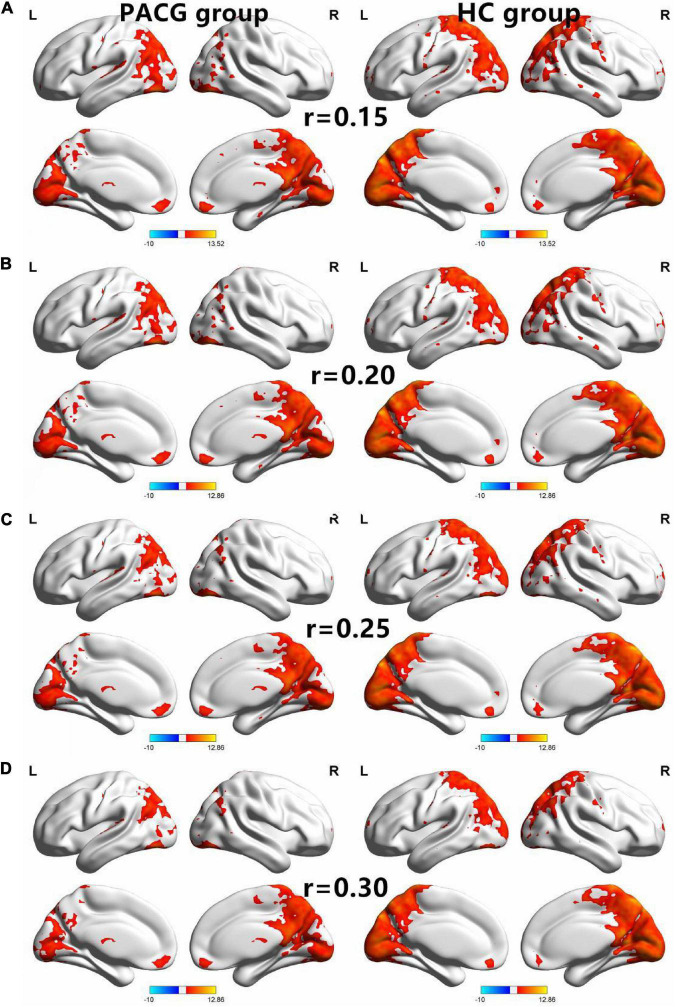
The spatial of the DC within the PACG and HC with different correlation thresholds (*r* = 0.15, 0.2, 0.25, 0.3). The group means DC maps of the PACG and HC [**(A)** (*r* = 0.15), **(B)** (*r* = 0.20), **(C)** (*r* = 0.25), and **(D)** (*r* = 0.30)]. PACG patients showed remarkably similar altered degree of centrality brain areas relative to healthy control in the different correlation thresholds (*r* = 0.15, 0.2, 0.25, 0.3) (FDR correction *p* < 0.001). DC, degree centrality; PACG, primary angle-closure glaucoma; HC, health control; L, left hemisphere; R, right hemisphere.

**FIGURE 2:**
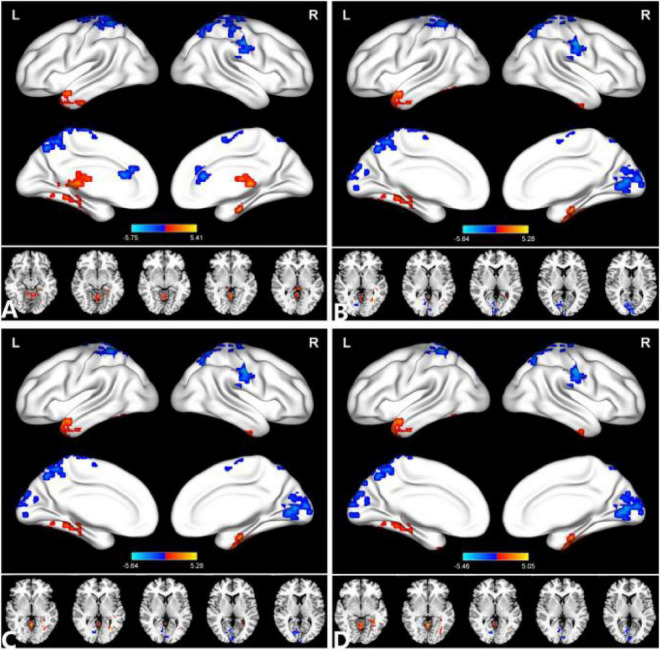
Comparison of the DC between the PACG and HC. **(A)** (*r* = 0.15), **(B)** (*r* = 0.20), **(C)** (*r* = 0.25), **(D)** (*r* = 0.30). Significant degree centrality differences were found between the two groups. The blue areas indicate lower degree of centrality values (two-tailed, voxel-level *P* < 0.01, GRF correction, cluster-level *P* < 0.05). GRF, Gaussian random field; L, left hemisphere; MOG, middle occipital gyrus; and R, right hemisphere.

The group means of DC maps of the PACG and HC [Figure 1A (*r* = 0.15) Figure 1B (*r* = 0.20), Figure 1C (*r* = 0.25) and Figure 1D (*r* = 0.3)]. Compared with HC, PACG patients had significantly higher DC values in the left cerebelum_Crus2, left cerebelum_4_5, right para hippocampal, and left thalamus. Meanwhile, PACG patients had significantly lower DC values in the right postcentral, left precuneus, and right postcentral (*r* = 015) (Table 2 and Figure 2A).

**TABLE 2:** Significant differences in the DC between the two groups (*r* = 0.15).

		MNI				
				
Condition	Brain regions	x	y	z	Peak *T*-scores	Cluster size (voxels)
*r* = 0.15						
PACG > HC	Cerebelum_Crus2_L	3	–51	–42	4.4289	178
PACG > HC	Cerebelum_4_5_L	0	–45	–18	5.4097	341
PACG > HC	ParaHippocampal_R	6	–15	–24	4.4358	44
PACG > HC	Thalamus_L	–6	–36	9	4.0191	56
PACG < HC	Postcentral_R	54	–15	39	–4.2974	76
PACG < HC	Precuneus_L	–12	–57	63	-5.7517	395
PACG < HC	Postcentral_R	39	–33	63	–4.4613	277

x, y, and z are the locations of the peak voxels in standard MNI coordinates.

DC, degree centrality; PACG: primary angle-closure glaucoma; HC: healthy control; MNI, Montreal Neurological Institute; R, right; L, left.

Compared with HC, PACG patients had significantly higher DC values in the right fusiform, left middle temporal gyrus, and left Cerebelum_4_5. Meanwhile, PACG patients had significantly lower DC values in the right calcarine, right postcentral gyrus, left precuneus gyrus, and left postcentral gyrus (*r* = 0.20) (Table 3 and Figure 2B).

**TABLE 3:** Significant differences in the DC between the two groups (*r* = 0.20).

		MNI				
				
Condition	Brain regions	x	y	z	Peak *T*-scores	Cluster size (voxels)
r = 0.20						
PACG > HC	Fusiform_R	39	–3	–39	3.8686	54
PACG > HC	Middle temporal gyrus_L	–39	9	–36	4.3994	67
PACG > HC	Cerebelum_4_5_L	0	–45	–18	5.2788	607
PACG < HC	Calcarine_R	3	–72	24	–4.4152	158
PACG < HC	Postcentral_R	54	–15	36	–4.4885	81
PACG < HC	Precuneus_L	–12	–57	63	–5.6443	120
PACG < HC	Postcentral_R	39	–33	63	–4.2005	198
PACG < HC	Postcentral_L	–27	–42	66	–4.2295	174

x, y, and z are the locations of the peak voxels in standard MNI coordinates.

DC, degree centrality; PACG, primary angle-closure glaucoma; HC, healthy control; MNI, Montreal Neurological Institute; R, right; L, left.

Compared with HC, PACG patients had significantly higher DC values in the right fusiform, left middle temporal gyrus, and left Cerebelum_4_5. Meanwhile, PACG patients had significantly lower DC values in the right calcarine, right postcentral gyrus, left precuneus gyrus, and left postcentral gyrus (*r* = 0.25) (Table 4 and Figure 2C).

**TABLE 4:** Significant differences in the DC between the two groups (*r* = 0.25).

		MNI				
				
Condition	Brain regions	x	y	z	Peak T-scores	Cluster size (voxels)
		*r* = 0.25				
PACG > HC	Fusiform_R	39	–3	–39	3.8686	54
PACG > HC	Middle temporal gyrus_L	–39	9	–36	4.3994	67
PACG > HC	Cerebelum_4_5_L	0	–45	–18	5.2788	607
PACG < HC	Calcarine_R	3	–72	24	–4.4152	158
PACG < HC	Postcentral_R	54	–15	36	–4.4885	81
PACG < HC	Precuneus_L	–12	–57	63	–5.6443	120
PACG < HC	Postcentral_R	39	–33	63	–4.2005	198
PACG < HC	Postcentral_L	–27	–42	66	–4.2295	174

x, y, and z are the locations of the peak voxels in standard MNI coordinates.

DC, degree centrality; PACG, primary angle-closure glaucoma; HC, healthy control; MNI, Montreal Neurological Institute; R, right; L, left,

Compared with HC, PACG patients had significantly higher DC values in the right fusiform gyrus, left middle temporal gyrus, left cerebelum_4_5, and right cerebelum_4_5. Meanwhile, PACG patients had significantly lower DC values in the right calcarine, right postcentral, left precuneus, left postcentral and left paracentral_lobule (*r* = 0.30) (Table 5 and Figure 2D).

**TABLE 5:** Significant differences in the DC between the two groups (*r* = 0.30).

		MNI				
				
Condition	Brain regions	x	y	z	Peak *T*-scores	Cluster size (voxels)
*r* = 0.30						
PACG > HC	Fusiform_R	39	–3	–39	3.7733	55
PACG > HC	Middle temporal gyrus_L	–39	9	–36	4.2944	75
PACG > HC	Cerebelum_4_5_L	0	–54	–3	5.0452	487
PACG > HC	Cerebelum_4_5_R	18	–39	–27	4.6504	47
PACG < HC	Calcarine_R	3	–72	24	–4.4721	187
PACG < HC	Postcentral_R	54	–15	36	–4.6369	85
PACG < HC	Postcentral_R	24	–30	75	–4.1747	178
PACG < HC	Precuneus_L	–12	–57	63	–5.4584	107
PACG < HC	Postcentral_L	–27	–42	66	–4.1125	86
PACG < HC	Paracentral_Lobule_L	–9	-27	78	–3.9634	57

x, y, and z are the locations of the peak voxels in standard MNI coordinates.

DC, degree centrality; PACG, primary angle-closure glaucoma; HC, healthy control; MNI, Montreal Neurological Institute; R, right; L, left.

### Support vector machine results

**FIGURE 3:**
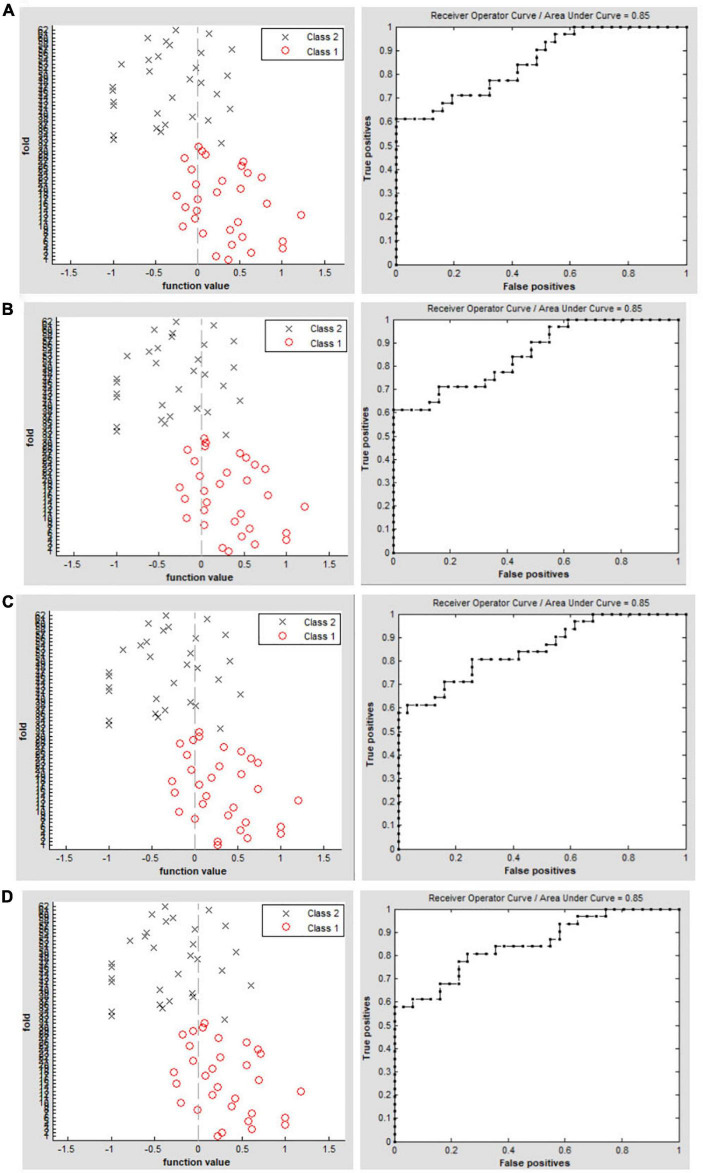
Classification results using machine learning analysis based on DC values. Function values of two groups (class 1: PACG group; class 2: HC group); The ROC curve of the SVM classifier with an AUC value of 0.85 (*r* = 0.15). **(A)** Function values of two groups (class 1: PACG group; class 2: HC group); the ROC curve of the SVM classifier with an AUC value of 0.85 (*r* = 0.20). **(B)** Function values of two groups (class 1: PACG group; class 2: HC group); The ROC curve of the SVM classifier with an AUC value of 0.85 (*r* = 0.25). **(C)** Function values of two groups (class 1: PACG group; class 2: HC group); The ROC curve of the SVM classifier with an AUC value of 0.85 (*r* = 0.30) **(D)**.

The SVM classification reaches a total accuracy of 72.58% and the ROC curve of the SVM classifier with an AUC value of 0.85 (*r* = 0.15); (Figure 3A). The SVM classification reaches a total accuracy of 75.81% and the ROC curve of the SVM classifier with an AUC value of 0.85 (*r* = 0.20); (Figure 3B). The SVM classification reaches a total accuracy of 74.19% and the ROC curve of the SVM classifier with an AUC value of 0.85 (*r* = 0.25); (Figure 3C). The SVM classification reaches a total accuracy of 77.42% and the ROC curve of the SVM classifier with an AUC value of 0.85 (*r* = 0.30); (Figure 3D).

## Discussion

In this study, compared with HCs, PACG patients had significantly higher DC values in the right fusiform gyrus, left middle temporal gyrus, and left cerebellum_4_5. Conversely, PACG patients had significantly lower DC values in the right calcarine sulcus, right postcentral gyrus, left precuneus, and left postcentral gyrus. The SVM classifier had an overall accuracy of 72.58%; the ROC curve of the SVM classifier had an AUC value of 0.85 (*r* = 0.25). These results demonstrated that PACG patients had widespread brain functional network hub dysfunction that affected the visual, auditory, default mode, and cerebellar networks. The findings might provide insights into the neural mechanism that underlies optic atrophy in PACG patients.

Notably, we found that PACG patients had significantly lower DC values in the right calcarine sulcus. The calcarine sulcus is a core component of the visual cortex; it plays an important role in transmitting visual information to the higher visual cortex. Previous neuroimaging studies showed visual dysfunction and vision-related cortex dysfunction in PACG patients. Colbert et al. (2021). found that glaucomatous mice with elevated intraocular pressure had decreased fractional anisotropy and increased radial diffusivity along the optic nerves and optic tract. Fujishiro et al. (2022) reported that ocular hypertension could lead to reduced cytochrome oxidase expression in the visual cortex. Pankowska et al. (2022) found that normal-tension glaucoma patients had reduced thickness in the right lateral occipital gyrus and left lingual gyrus. Consistent with these findings, we observed significantly lower DC values in the right calcarine sulcus in PACG patients, which suggested abnormal functional connectivity within the visual network.

Another important finding in this study was that PACG patients had significantly lower DC values in the right postcentral gyrus and left postcentral gyrus. The postcentral gyrus has an important role in sensorimotor function. There are two possible reasons for the phenomenon observed in our study. First, PACG patients exhibit visual loss associated with optic atrophy. Using diffusion tensor MRI, Yang et al. (2018) demonstrated that glaucomatous mice had extensive deterioration of visuomotor function by 9 months of age. Zwierko et al. (2022) reported differences in visuomotor task performance among older adults with moderate and advanced glaucoma. Dive et al. (2016) demonstrated visuomotor behavior in patients with glaucoma-related peripheral field reduction during the completion of natural movements. Thus, decreased DC values in the postcentral gyrus might indicate visuomotor dysfunction in PACG patients. Second, the postcentral gyrus plays an important role in nociceptive processing. Glaucoma patients experience painful swelling in both eyes. Dong et al. (2019) demonstrated that patients with eye pain had decreased voxel-mirrored homotopic connectivity in the precentral/postcentral gyrus. Additionally, Pan et al. (2018) reported that eye pain patients had a lower amplitude of low-frequency fluctuation in the left and right precentral/postcentral gyrus and left precuneus. Consistent with these findings, our study revealed that PACG patients had lower DC values in the postcentral gyrus, which might reflect visuomotor dysfunction and eye pain in PACG patients.

Finally, we found that PACG patients had significantly lower DC values in the left precuneus and increased DC values in the left middle temporal gyrus. The precuneus and middle temporal gyrus are core components of the default mode network (DMN). The DMN is an endogenous neural network that shows consistently higher blood oxygenation level-dependent activity during rest. It plays an important role in self-referential thought and introspection; these actions involve various higher cognitive functions such as memory, prospection, and self-processing (Raichle et al., 2001). Wang et al. (2017) demonstrated that POAG patients had decreased functional connectivity in the DMN. Giorgio et al. (2020) reported that ocular hypertension patients had decreased functional connectivity in key cognitive networks [DMN and frontoparietal working memory network (WMN)]. Our finding of significantly lower DC values in the DMN of PACG patients suggests that those patients are experiencing cognitive decline.

In our study, we used the DC maps as a feature. The SVM model was applied to investigate the SVM classification reaches a total accuracy of 72.58%–77.42% and the ROC curve of the SVM classifier with an AUC value of 0.85. The SVM modal showed a high sensitivity for distinguishing the two groups. The DC maps could be sensitive biomarkers for distinguishing patients with PACG from HCs.

Some limitations should be mentioned in the study. First, the sample size of the study is relatively small. Second, the DC values are based on blood oxygen levels dependent (BOLD) on signals, which might be affected by physiological noise. Third, no multimodal MRI methods were used to verify the results of this study.

## Conclusion

Our results showed that PACG patients showed widespread brain functional network hub dysfunction relative to the visual network, auditory network, default mode network, and cerebellum network, which might shed new light on the neural mechanism of optic atrophy in PACG patients. Thus, the DC maps could be sensitive biomarkers for distinguishing patients with PACG from HCs. It provides an important imaging reference for clinicians in early diagnosis.

## Data availability statement

The raw data supporting the conclusions of this article will be made available by the authors, without undue reservation.

## Ethics statement

The studies involving human participants were reviewed and approved by the Medical Ethics Committee of the Jiangxi Provincial People’s Hospital. The patients/participants provided their written informed consent to participate in this study.

## Author contributions

R-BC, Y-LZ, HL, and XH contributed to data collection and statistical analyses, wrote the manuscript, designed the protocol, contributed to the MRI analysis, designed the study, oversaw all the clinical aspects of study conduct, and prepared the manuscript. All authors contributed to the article and approved the submitted version.

## Footnotes

1
http://www.rfmri.org/dpabi
